# Intra- and interfamily phenotypic diversity in pain syndromes associated with a gain-of-function variant of Na_V_1.7

**DOI:** 10.1186/1744-8069-7-92

**Published:** 2011-12-02

**Authors:** Mark Estacion, Chongyang Han, Jin-Sung Choi, Janneke GJ Hoeijmakers, Giuseppe Lauria, Joost PH Drenth, Monique M Gerrits, Sulayman D Dib-Hajj, Catharina G Faber, Ingemar SJ Merkies, Stephen G Waxman

**Affiliations:** 1 Department of Neurology, Yale University School of Medicine, New Haven, CT 06510, and Center for Neuroscience and Regeneration Research, Veterans Affairs Medical Center, West Haven, CT 06516, USA; 2 Department of Neurology, University Medical Center Maastricht, Maastricht, the Netherlands; 3 Neuromuscular Diseases Unit, IRCCS Foundation, "Carlo Besta", Milan, Italy; 4 Department of Gastroenterology and Hepatology, Radboud University Nijmegen Medical Center, Nijmegen, the Netherlands; 5 Department of Clinical Genetics, University Medical Center Maastricht, Maastricht, the Netherlands; 6 Department of Neurology, Spaarne Hospital, Hoofddorp, the Netherlands; 7 College of Pharmacy, Catholic University of Korea, Bucheon, South Korea

## Abstract

**Background:**

Sodium channel Na_V_1.7 is preferentially expressed within dorsal root ganglia (DRG), trigeminal ganglia and sympathetic ganglion neurons and their fine-diamter axons, where it acts as a threshold channel, amplifying stimuli such as generator potentials in nociceptors. Gain-of-function mutations and variants (single amino acid substitutions) of Na_V_1.7 have been linked to three pain syndromes: Inherited Erythromelalgia (IEM), Paroxysmal Extreme Pain Disorder (PEPD), and Small Fiber Neuropathy (SFN). IEM is characterized clinically by burning pain and redness that is usually focused on the distal extremities, precipitated by mild warmth and relieved by cooling, and is caused by mutations that hyperpolarize activation, slow deactivation, and enhance the channel ramp response. PEPD is characterized by perirectal, periocular or perimandibular pain, often triggered by defecation or lower body stimulation, and is caused by mutations that severely impair fast-inactivation. SFN presents a clinical picture dominated by neuropathic pain and autonomic symptoms; gain-of-function variants have been reported to be present in approximately 30% of patients with biopsy-confirmed idiopathic SFN, and functional testing has shown altered fast-inactivation, slow-inactivation or resurgent current. In this paper we describe three patients who house the Na_V_1.7/I228M variant.

**Methods:**

We have used clinical assessment of patients, quantitative sensory testing and skin biopsy to study these patients, including two siblings in one family, in whom genomic screening demonstrated the I228M Na_V_1.7 variant. Electrophysiology (voltage-clamp and current-clamp) was used to test functional effects of the variant channel.

**Results:**

We report three different clinical presentations of the I228M Na_V_1.7 variant: presentation with severe facial pain, presentation with distal (feet, hands) pain, and presentation with scalp discomfort in three patients housing this Na_V_1.7 variant, two of which are from a single family. We also demonstrate that the Na_V_1.7/I228M variant impairs slow-inactivation, and produces hyperexcitability in both trigeminal ganglion and DRG neurons.

**Conclusion:**

Our results demonstrate intra- and interfamily phenotypic diversity in pain syndromes produced by a gain-of-function variant of Na_V_1.7.

## Introduction

Sodium channel Na_V_1.7 is preferentially and abundantly expressed within dorsal root ganglia (DRG) [1,2], trigeminal ganglia [3] and sympathetic ganglion neurons [1,2], and their fine-diameter axons [4]. The physiological attributes of Na_V_1.7 include slow closed-state inactivation, which permits activation of the channel in response to small, slow depolarizations close to resting potential [5]. Na_V_1.7 thus acts as a threshold channel, amplifying stimuli such as generator potentials in nociceptors, thereby setting their gain [6].

Gain-of-function mutations and variants (single amino acid substitutions) of Na_V_1.7 have been linked to three pain syndromes. Inherited erythromelalgia (IEM) is characterized clinically by burning pain and redness that is usually focused on the distal extremities, precipitated by mild warmth and relieved by cooling, and is caused by Na_V_1.7 mutations that hyperpolarize activation, slow deactivation, and enhance the channel ramp response [7]. Paroxysmal extreme pain disorder (PEPD) is characterized by perirectal, periocular or perimandibular pain, often triggered by defecation or lower body stimulation [8], and has been linked to Na_V_1.7 mutations that severely impair fast-inactivation [9]. Small Fiber Neuropathy (SFN), which involves thinly myelinated and unmyelinated peripheral nerve fibers [10,11], presents a clinical picture that is characteristically dominated by neuropathic pain and autonomic symptoms [12], together with preservation of normal strength, tendon reflexes, and vibration sense, and normal nerve conduction studies (NCS), which rule out large fiber involvement. The diagnosis of SFN can be confirmed by demonstration of reduced intraepidermal nerve fiber density (IENFD) on skin biopsy and/or abnormal quantitative sensory testing (QST) [13,14]. No apparent cause for SFN can be identified in 24% to 93% of cases in published patient series, and these cases are termed idiopathic I-SFN [10,15,16]. Faber *et al.*, recently reported that gain-of-function variants (single amino acid substitutions) of voltage-gated sodium channel Na_V_1.7 are present in approximately 30% of patients with biopsy-confirmed I-SFN [17].

Distal (feet, and in some cases, hands) burning or stabbing pain or paraesthesias are the initial symptoms in most patients with I-SFN, and facial pain is rare. Most of the eight patients with SFN described earlier by Faber *et al.*, [17] fit this clinical picture, and presented with pain in the feet and in some cases the hands early in their course, but did not manifest facial pain [17]. In contrast, one patient in this series presented with severe pain in the teeth, jaw, and behind the eyes. This patient (patient 8 in Faber *et al.*, 2011) harbored the Na_V_1.7 variant c.684C > G (I228M) [17]; functional properties of this variant have not been previously reported. We subsequently studied the sister of this patient, who houses the same variant (c.684C > G (I228M) in Na_V_1.7) and suffers from a different syndrome of pain and redness of the hands and feet triggered by warmth, and have encountered an additional patient housing the same Na_V_1.7 variant with pain over the scalp. In this study we report these three different clinical presentations of the I228M Na_V_1.7 variant, and demonstrate the effects of the Na_V_1.7/I228M channels on excitability in both trigeminal ganglion and DRG neurons.

## Results

### Patient 1

This patient, described as patient number 8 in Faber *et al.*, (2011) [17] is a 51-year-old male, referred with complaints that started at age 32, when he experienced excruciating pain in his teeth and jaw triggered by cold and heat, which could radiate to the temporomandibular joint, and pain behind both eyes, especially when looking at bright light. The oral mucosa, lips and tongue were not affected. Multiple tooth extractions did not provide pain relief. He subsequently developed myalgia, with muscle pain persisting for 5-6 days after light physical activity. The pain was aggravated by cold temperature and relieved by warmth. Sometimes the feet were also swollen. This patient suffered from stomach cramps and diarrhea for more than 35 years, and from dry mouth and eyes and reduced urinary sensation and intermittent hesitation for several years. The patient was severely disabled and unable to work due to these complaints. Acetaminophen made the pain bearable, while short trials of NSAIDs and antidepressants did not provide relief. Physical examination showed no abnormalities. Laboratory investigations, nerve conduction studies and chest X-ray were normal. IENFD (1.6/mm; age- and gender-matched normal values ≥ 3.5/mm [18]) was abnormal. QST revealed abnormal warm and cold thresholds of the right foot. *SCN9A *gene analyses demonstrated the variant, c.684C > G; Na_V_1.7/I228M. The patient was diagnosed with Na_V_1.7-related SFN. The patient's two sons, aged 27 and 29, were found to house the I228M substitution, but did not have any complaints at the time of study.

**Figure 1:**
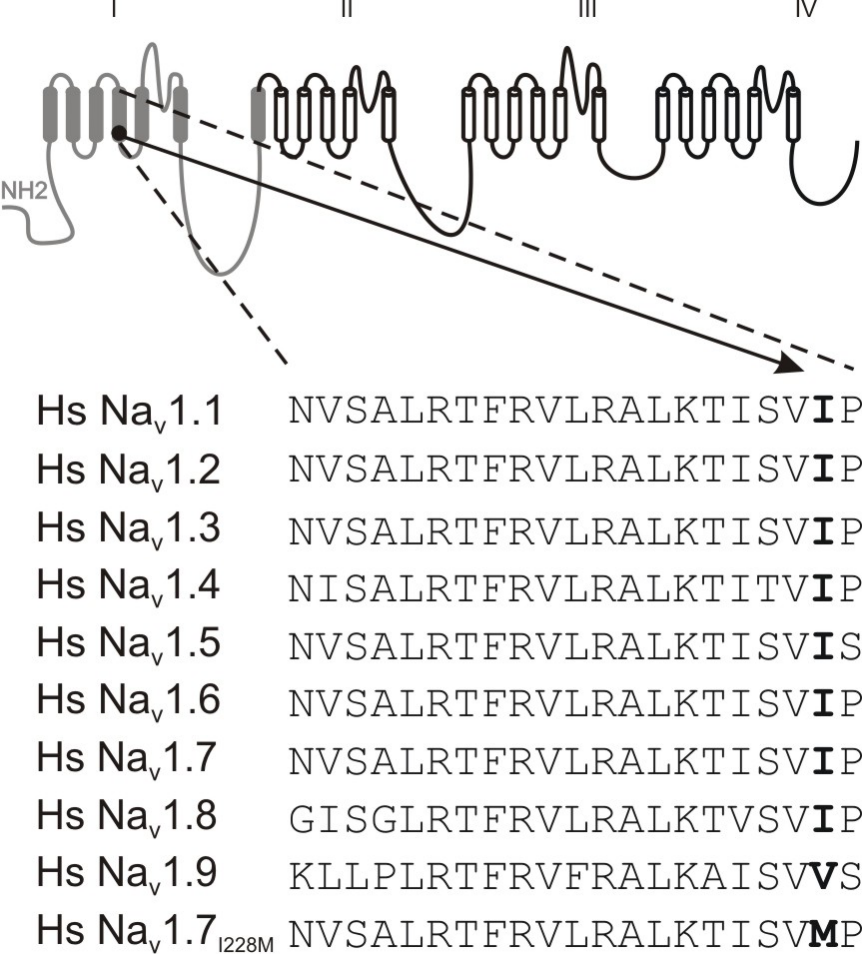
**Schematic of I228M mutation**. Sequence alignment of DI/S4 from human sodium channels. The charge-conserved substitution in DI/S4 replaces a highly conserved isoleucine residue at the cytoplasmic end of the S4 helix. I228 is conserved in all human sodium channels except for Na_V_1.9 which has a conservative substitution, valine, at the corresponding position.

The I228M variant substitutes a highly conserved residue near the C-terminus of the S4 segment in domain I (DI/S4, Figure 1). All human sodium channels except Na_V_1.9 carry an isoleucine at this position [19], and this residue is invariant in all Na_V_1.7 orthologues from mammalian species (data not shown). The conservation of the I228 residue among sodium channels suggests that the I228M substitution might alter the properties of the Na_V_1.7 channels (Figure 1). This substitution was not found in a control panel of DNA from 100 healthy Dutch (Caucasian) individuals (200 chromosomes). However, I228M is listed as a natural SNP in one database (Craig Venter Human Genome), but with no breakdown of major/minor allele frequency, and has been reported as being associated with Dravet syndrome [20] and in < 0.3% of a control population (5/576 control chromosomes).

### Patient 2

This patient, who is the sister of Patient 1, gave a history of burning pain and redness of hands and feet, triggered by rising temperature and exercise and relieved by cooling, beginning at age 36 years. She also reported increased perspiration, gastrointestinal complaints and hot flashes. Her medical history revealed recurrent urticaria attacks, psoriatic arthritis and hypothyroidism for which she is adequately treated. Physical examination showed no abnormalities other than red discolored hands. Laboratory investigations, nerve conduction studies and chest X-ray were normal. Quantitative sensory testing showed no abnormalities. IENFD was 8 per mm (normal values ≥ 5.7 per mm) [18]. DNA analysis showed the same substitution in the *SCN9A *gene as found in her brother. On the basis of the clinical history and findings, the patient was diagnosed as having probable SFN.

### Patient 3

This 46-year-old woman presented with a red discoloration of the occiput. Three months later the red area expanded and was noted to be associated with a tingling, burning and warm sensation over the scalp. After washing of the hair, the redness increased for ~one hour. The patient also complained that the structure of her hair changed, becoming dryer and more fragile. She noted improvement in these complaints with warm temperatures (such as during a visit to the Caribbean) and with fever. Cold had no specific influence. A year following onset of scalp symptoms, a red discoloration of the toes of both feet developed, together with paraesthesias and a burning sensation and tingling in both hands. In addition, the patient reported severe perspiration since puberty and intermittent difficulties with micturition. No other dysautonomic symptoms were noted. Ibuprofen did not relieve the pain. The family history was negative. Neurological examination was unremarkable. Laboratory investigations, a chest X-ray and nerve conduction studies were normal. Quantitative sensory testing showed abnormal thresholds for warmth and cold sensation of the dorsum of the right foot. A skin biopsy demonstrated an INFD of 5.2 per mm, which was lower than the reported normative values (≤ 5.7/mm) [18]. The patient was diagnosed as having I-SFN. *SCN9A *gene analysis demonstrated the same variant, c.684C > G; p. I228M, as in Patients 1 and 2.

## Functional Analysis

### Voltage-clamp analysis

**Figure 2:**
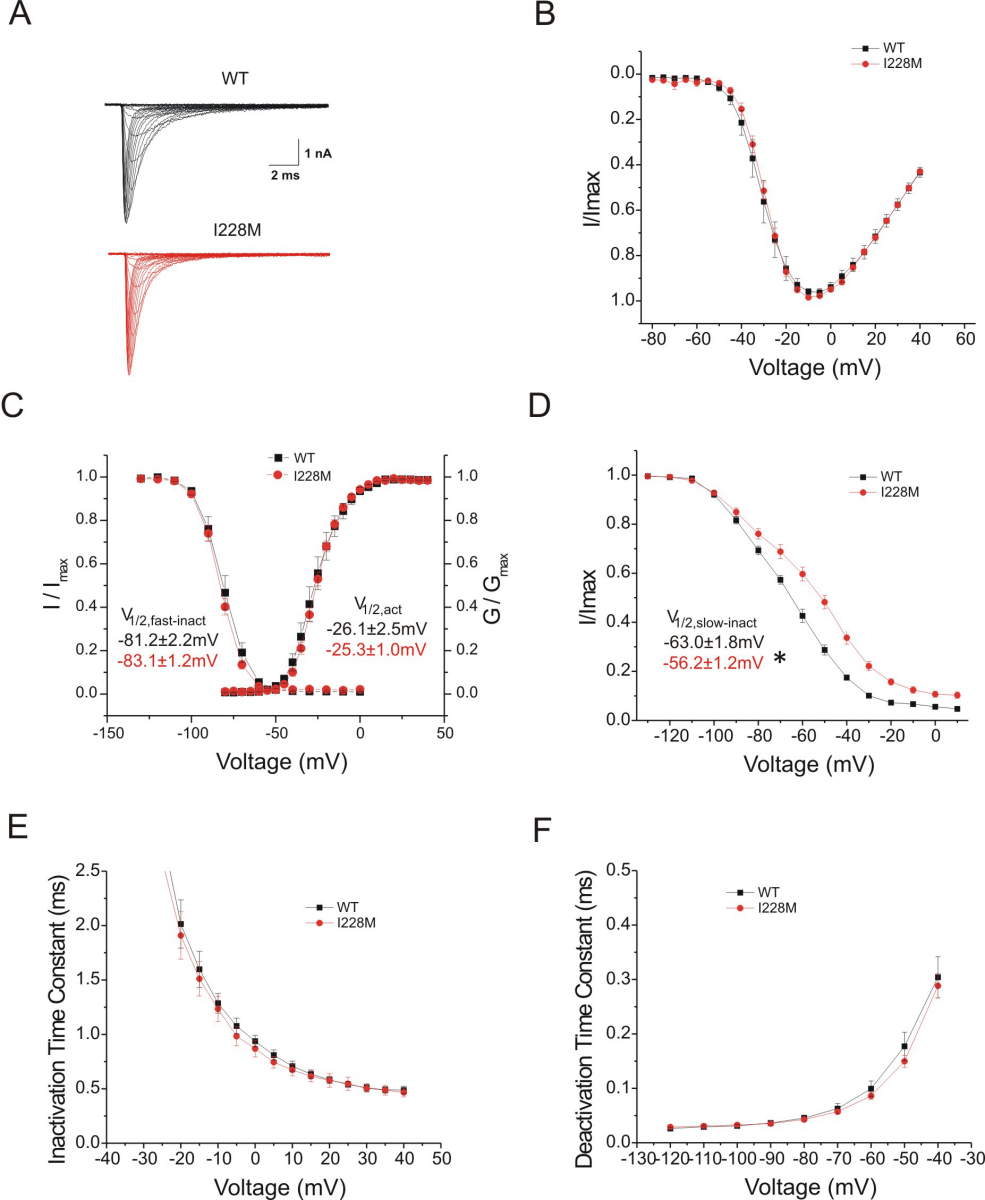
**Voltage-clamp properties ofNa_V_1.7/I228Mchannels in HEK293 cells**. Electrophysiological analysis of I228M variant: (A) Representative current traces recorded from HEK293 cells expressing wild type Nav1.7 (WT) (top) or I228M (bottom) channels, evoked by voltage steps (100 mec) from -80 to +40 mV in 5 mV increments, from a holding potential of -120 mV. (B) Normalized I-V curves for WT and I228M expressing cells. (C) Activation and steady-state fast-inactivation for WT (black squares) and I228M (red circles). Fast-inactivation was examined using a series of 500 msec prepulses from -140 to 0 mV followed by test pulses to -10 mV. Left inset: midpoint values for fast inactivation (V_1/2, fast-inact_) of WT (black) and I228M (red). Right inset: midpoint values for activation (V_1/2, act_) of WT (black) and I228M (red). (D) Steady-state slow-inactivation of WT (black squares) and I228M (red circles). Slow-inactivation was assessed using a 20 msec pulse to -10 mV after a 30 second prepulse to potentials from -130 to 10 mV followed by a 100 msec pulse to -120 mV to remove fast-inactivation. Inset: midpoint values of slow-inactivation (V_1/2, slow-inact_) (WT: black; I228M: red); *p < 0.05. V_1/2 _represents voltage midpoint, I/I_max_represents normalized current, and G/G_max _represents normalized conductance for fast-activation, slow-inactivation, and activation. (E) The kinetics of inactivation were analyzed by fitting data with a single exponential function for WT and I228M currents. (F) The kinetics of deactivation for WT and I228M expressing cells were obtained by holding the cells at -120 mV and tail currents were generated by a brief 0.5 ms depolarization to -20 mV followed by a series of repolarizations ranging from -120 to -40 mV. The closing rate of the channels was obtained by fitting the tail currents with a single exponential function.

Voltage-clamp analysis of I228M variant channels following expression in HEK293 cells (Figure 2) demonstrated impaired slow-inactivation (Figure 2D). Current densities (WT: 432 ± 90 pA/pF, n = 9; I228M: 357 ± 70 pA/pF n = 13), activation V_1/2 _(WT: -26.1 ± 2.5 mV, n = 9; I228M: -25.3 ± 1.0 mV, n = 13), and fast-inactivation V_1/2 _(WT: -81.2 ± 2.2 mV, n = 8; I228M: -83.1 ± 1.2 mV, n = 12), for HEK293 cells transfected with WT or I228M channels were not significantly different (Figure 2A, B, C). The time constants for fast-inactivation (Figure 2E) and deactivation (Figure 2F) were not significantly different for I228M versus WT channels. Persistent current (non-inactivating component at 0 mV), measured in CsF-based pipette solution (WT: 0.42% ± 0.12%, n = 8; I228M: 0.67% ± 0.20%, n = 12) and in aspartate-based pipette solution (WT: 0.41% ± 0.08%, n = 14; I228M: 0.41% ± 0.15%, n = 13) were not significantly different for I228M versus wild-type channels. Slow-inactivation was impaired for I228M channels (Figure 2D), with a depolarized V_1/2 _(WT: -63.0 ± 1.8 mV, n = 10; I228M: -56.2 ± 1.2 mV, n = 14; p < 0.05). The offset for slow-inactivation (non-inactivating component at 10 mV) was not significantly larger for I228M compared to wild-type channels (WT: 7.8% ± 1.3%, n = 10; I228M: 7.8% ± 1.3%, n = 14). Impaired slow-inactivation would be expected to increase the number of channels available for activation at potentials positive to -100 mV, including potentials close to resting potential of DRG neurons.

### Current-Clamp Analysis: DRG Neurons

**Figure 3:**
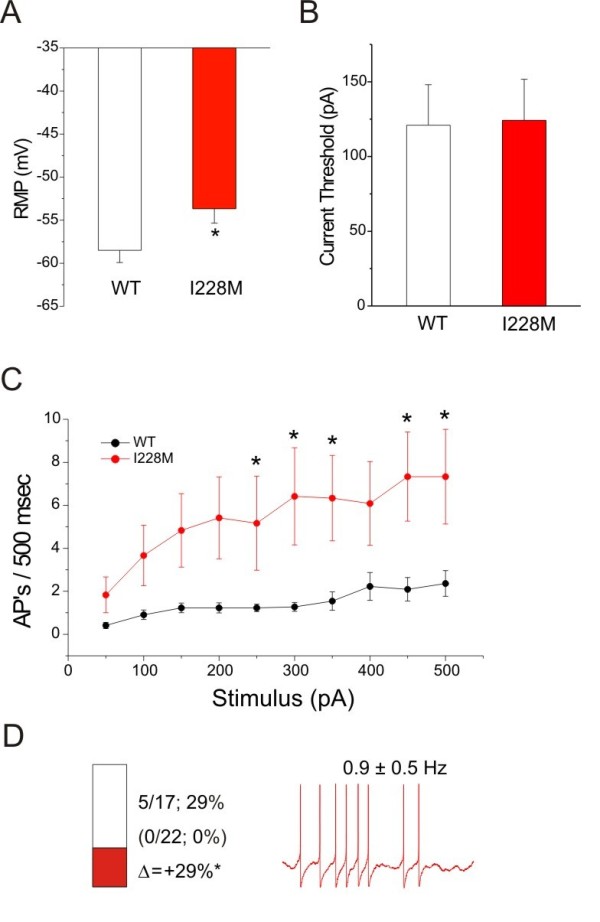
**Current-clamp properties of DRG neurons transfected with I228M. **Excitability of DRG neurons expressing I228M: (A) RMP of DRG neurons expressing WT (-58.5 ± 1.4, n = 22) or I228M (-53.7 ± 1.7, n = 12); *p < 0.05. (B) Current threshold of DRG neurons expressing WT (121 ± 27, n = 22) or I228M (124 ± 28, n = 12) to 200 msec stimuli. (C) Comparison of mean firing frequency in DRG neurons expressing WT and I228M across a range of current injections from 50 to 500pA; *p < 0.05. (D) Bar graph showing the proportion of spontaneous firing cells for DRG neurons expressing I228M (red) and WT channels (black); numbers to the right of the bar graph show values for WT (lower value in parentheses) and I228M (upper value); *p < 0.05. The recording on the right shows spontaneous firing (10 seconds) of representative DRG neuron expressing I228M; the numbers above the trace show average ± standard deviation frequency of spontaneous action potentials. APs = action potentials.

I228M had strong functional effects on DRG neurons, which were clearly rendered hyperexcitable by these channels (Figure 3). I228M produced a 4.8 mV depolarizing shift in resting membrane potential of transfected neurons (WT: -58.5 ± 1.4 mV, n = 22; I228M: -53.7 ± 1.7 mV, n = 12; p < 0.05). While I228M did not decrease the current threshold (WT: 121 ± 27 pA, n = 22; I228M: 124 ± 28 pA, n = 12), it produced on average a higher firing frequency at all stimulus intensities, even close to current threshold, and increased the number of action potentials evoked by 500-millisecond depolarizing stimuli at higher stimulus intensities nearly four-fold, with the change being statistically significant at many of the intensities tested, from 50 to 500 pA. I228M also produced a significant increase in the proportion of spontaneously firing cells (5 of 17 [29%] vs 0 of 22 [0%] for cells transfected with WT channels) (p < 0.05); mean frequency of spontaneous activity in cells transfected with I228M was 0.9 ± 0.5 Hz (n = 5).

### Current Clamp Analysis: Trigeminal Ganglion Neurons

**Figure 4:**
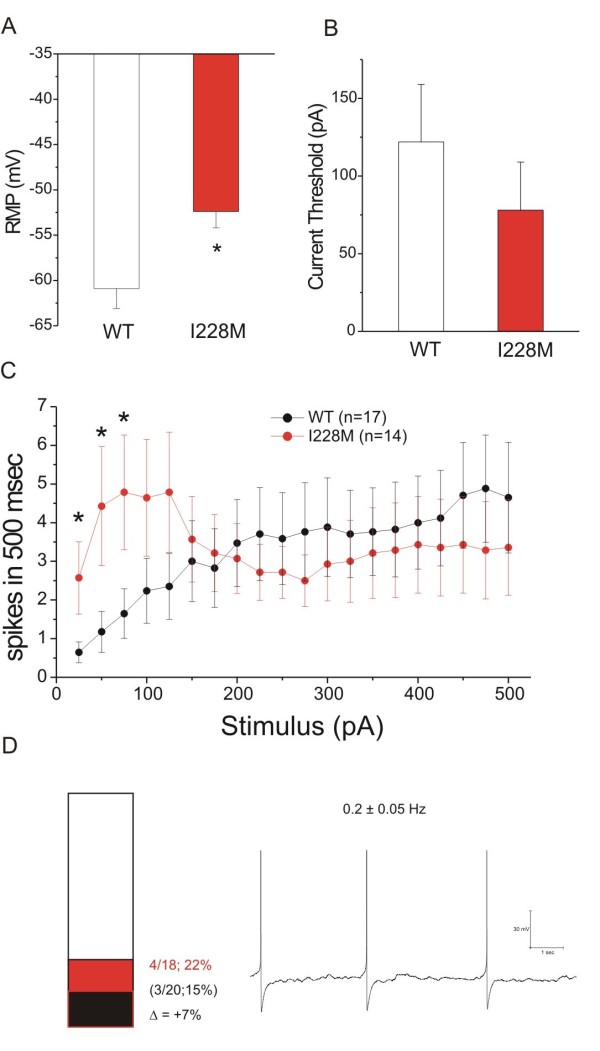
**Current-clamp properties of trigeminal ganglion neurons transfected with I228M**. Excitability of trigeminal ganglion neurons expressing I228M: (A) RMP of trigeminal ganglion neurons expressing WT (-60.9 ± 2.2, n = 17) or I228M (-52.4 ± 1.8, n = 14); *p < 0.05. (B) Current threshold of DRG neurons expressing WT (122 ± 37, n = 13) or I228M (78 ± 31, n = 12) to 200 msec stimuli; p < 0.05. (C) Comparison of mean firing frequency in trigeminal ganglion neurons expressing WT and I228M across a range of current injections from 25 to 500 pA; *p < 0.05. (D) Bar graph showing the proportion of spontaneous firing cells for trigeminal neurons expressing I228M (red) and WT channels (black); numbers to the right of the bar graph show mean values for WT (lower value in parentheses) and I228M (upper value). The recording on the right shows spontaneous firing (10 seconds) of representative trigeminal neuron expressing I228M; the numbers above the trace show average ± standard deviation frequency of spontaneous action potentials. APs = action potentials.

The I228M variant produced hyperexcitability in trigeminal ganglion neurons (Figure 4). I228M produced an 8.5 mV depolarizing shift in resting membrane potential (WT: -60.9 ± 2.2 mV, n = 17; I228M: -52.4 ± 1.8 mV, n = 14; p < 0.05). I228M produced a 36% reduction in current threshold to 200-millisecond stimuli (WT: 122 ± 37 pA, n = 13; I228M: 78 ± 31 pA, n = 12). Trigeminal ganglion neurons transfected with I228M tended to fire multiple action potentials (at a frequency nearly four-fold higher than in cells transfected with wild-type channels) in response to 500 msec stimuli close to threshold (25 to 125 pA, with the difference being statistically significant between 25 and 75 pA), although at stimulus levels of ≥ 2X threshold, the number of action potentials falls, approaching that of cells transfected with wild-type channels (Figure 4C). I228M produced a trend toward an increase in the proportion of spontaneously firing cells (4 of 18 [22%] vs 3 of 20 [15%] for cells transfected with WT channels) that did not reach statistical significance; mean frequency of spontaneous activity in cells transfected with I228M was 0.2 ± 0.05 Hz (n = 4).

## Discussion

In this study we describe three patients (two siblings, and a third, unrelated patient) housing the I228M variant of sodium channel Na_V_1.7. One of these patients displayed a clinical phenotype that included pain in the face as well as in other parts of the body together with autonomic symptoms, with the diagnosis of SFN confirmed by demonstration of reduced IENFD on skin biopsy, and abnormal QST. The second patient gave a history of distal extremity pain and redness, triggered by warmth and relieved by cooling. While these symptoms are commonly reported in IEM [7,21], she also reported autonomic symptoms including increased perspiration, gastrointestinal complaints and hot flashes, which are not characteristic of IEM. The third patient initially experienced discomfort and vasomotor instability over the occiput, which progressed to involve the distal extremities, together with abnormal perspiration, intermittent difficulties with micturition; skin biopsy and QST in this patient were both abnormal, confirming the diagnosis of SFN.

Because facial pain was a prominent part of the clinical picture in one of the patients described in this paper, we assessed the effect of the I228M mutation on excitability of trigeminal ganglion neurons. Our current-clamp analysis demonstrated that the I228M variant depolarizes resting membrane potential, reduces current threshold and enhances repetitive firing in these cells. The effect of only one other Na_V_1.7 mutation has been assessed in trigeminal ganglion neurons. We previously reported that the A1632E Na_V_1.7 mutation, from a patient who displayed a mixed clinical phenotype with features of both IEM and PEPD, produces hyperexcitability in trigeminal ganglion neurons [22]. The A1632E mutation, however, produced hyperexcitability in these cells over the entire range of stimulus intensities, while I228M produces hyperexcitability only at low stimulus intensities. Whether other gain-of-function mutations of Na_V_1.7 have similar effects on trigeminal ganglion neurons remains to be determined.

The I228M substitution is located within the fourth transmembrane segment (S4) within domain I of the Na_V_1.7 channel. The S4 in each of the domains of sodium channels is an amphiphatic helix which is characterized by a repeat motif of positively charged amino acids at every third position [19]. Non-charge-conserved mutations, S211P and F216S, in DI/S4 have been linked to IEM, and have been shown to shift voltage-dependence of activation in a hyperpolarizing direction, making it easier to open the mutant channels [23,24]. The I228M substitution does not change the number of charges in the S4 segment, and reasonably conserves the hydrophobic nature of the side-chain of this residue, and thus might not have been predicted to have a functional effect. A link to function, however, is suggested by the conservation of the I228 residue at the equivalent position in all voltage-gated sodium channels sequenced to date (Figure 1); I228 is substituted by the other branched side-chain residue, valine, in Na_V_1.9. The functional effect of I228M might be related to the proximity of the I228 residue to the cytoplasmic end of the S4 segment, which could alter the local structure of the helix in a subtle manner affecting slow-inactivation but not activation. Notably, while the I228M variant produced hyperexcitability in both DRG and trigeminal ganglion neurons, only two of the three patients described here reported cranial pain, and it was experienced in the jaw and eyes in one, while it was focused on the scalp in the other.

Our results demonstrate phenotypic diversity in the pain syndromes associated with the I228M substitution in the Na_V_1.7 channel in three different patients. Two of these patients were from the same family, which also includes patient 1's two asymptomatic sons who carry the I228M Na_V_1.7 variant. Both of these asymptomatic carriers are younger than the age of onset of the three patients presented, and whether they will develop pain in the future is unclear. We have previously noted different ages of onset and different degrees of pain, and an asymptomatic carrier in members of a single family, all housing the G616R Na_V_1.7 mutation [25]. Whether this phenotypic variability is due to modifier genes, epigenetic factors, and/or environmental factors is not yet clear. The minor allele of the Na_V_1.7 R1150W variant, which is known to produce hyperexcitability in DRG neurons [26], has been associated with increased pain scores in a number of acquired pain syndromes (osteoarthritis, compressive radiculopathies, traumatic limb amputation), suggesting that environmental factors may, at least in some individuals, act as triggers or increase risk of developing pain [27].

Most peripheral neuropathies present in a "stocking glove" distribution with sensory abnormalities and pain first appearing in the most distal parts of the limbs (feet, then hands). It has traditionally been held that longer nerve fibers, or the cells giving rise to them, are affected before shorter fibers or the cells giving rise to them. A number of potential mechanisms have been invoked for this length-dependent mode of progression of neuropathy, including impairment of axoplasmic transport [28], increased probability of demyelination along longer nerve fibers [29], or a higher probability of impairment of calcium homeostasis along longer nerve fibers [30,31]. However, the present results show that the Na_V_1.7 I228M variant, which impairs slow-inactivation, produces physiological changes in primary afferent neurons (trigeminal ganglion neurons) that innervate the relatively proximal sensory field of the face and scalp, as well as DRG neurons. While we do not know whether there was degeneration of small fibers innervating the face or scalp in these patients, both exhibited degeneration of the relatively long axons, as demonstrated by reduced IENFD on skin biopsy from the leg.

In summary, our results demonstrate phenotypic diversity in pain syndromes associated with the I228M gain-of-function variant of Na_V_1.7. Importantly, variability in clinical presentation was present not only when comparing patients from different families, but also for patients within a single family. Our findings also demonstrate that the I228M variant can increase excitability of trigeminal ganglion as well as DRG neurons. While the mechanism(s) responsible for this phenotypic diversity remain unexplained, our findings suggest that clinical studies, in patients who are carriers of functional variants of sodium channels, should be designed to take phenotypic variability, even within single families, into account.

## Materials and methods

### Patients

The three patients initially studied were part of a cohort of patients aged ≥ 18 years with idiopathic SFN, seen at Maastricht University Medical Center Neurological Clinic, with a clinical diagnosis of SFN between 2006 and 2009; this series excluded patients in whom, after a careful work-up, a cause for SFN was identified. A sister of patient 1 was also studied. This study was approved by medical ethics committees at Yale University and Maastricht University Medical Center. All aspects of the study were explained and a written informed consent obtained prior to study.

All three patients met strict eligibility criteria for a study on SFN as described by Faber et al. [17]. Subjects were excluded from the study if there was a history or detection after screening of illnesses known to cause SFN, including impaired glucose tolerance, diabetes mellitus, hyperlipidemia, liver/kidney/thyroid dysfunction, monoclonal-gammopathy, connective tissue disorders, amyloidosis, sarcoidosis, Fabry's disease (alpha-galactosidase, in females combined with GLA-gene sequencing), celiac disease, HIV, alcohol abuse, hemochromatosis, B6 intoxication, anti-phospholipid syndrome neurotoxic drugs (e.g., chemotherapy) [17].

### Clinical characterization

#### Skin biopsy

Punch biopsy (10 cm above lateral malleolus) specimens were fixed (2% paraformaldehyde-lysine-sodium periodate at 4°C), cryprotected and stored at -80°C in 20% glycerol before sectioning (50 μm) [13]. The numbers of individual nerve fibers crossing the dermal-epidermal junctions were analyzed by bright-field microscopy (Olympus BX50 stereology workstation, PlanApo oil-objective 40×/NA = 1.0) in each of three sections, immunostained with polyclonal rabbit antiprotein-gene-product-9.5 antibody (PGP9.5; Ultraclone, Wellow, Isle-of-Wight, UK). Linear quantification of intraepidermal nerve fiber density (IENF/mm) was compared with age- and gender-adjusted normative values [18,32].

#### Quantitative sensory testing (QST)

QST, performed in accordance with previous guidelines [33], using a TSA-2001 (Medoc, Ramat-Yishai, Israel) instrument, assessed thresholds at the dorsum of both feet and thenar eminences, using ascending/descending (warm/cool) thermal ramp stimuli delivered through a thermode [34]. Heat pain modality was also examined. Results were compared with reported normative values [35]. Measurements were considered abnormal when Z-values exceeded 2.5. A sensory modality was classified as abnormal if results of both method-of-limits and method-of-levels were abnormal [36].

### SCN9A sequence analysis

#### Exon screening

Genomic DNA was extracted from 300 μL whole blood using Puregene genomic DNA isolation kit (Gentra-Systems, Minneapolis). All *SCN9A *coding exons and flanking intronic sequences, and exons encoding 5' and 3-untranslated sequences within the complementary DNA, were amplified and sequenced as described previously [37]. Genomic sequences were compared with reference Na_v_1.7 cDNA (NM_002977.3) to identify sequence variations [38] using Alamut Mutation-Interpretation Software (Interactive-Biosoftware; Rouen, France). A control panel of DNA from 100 healthy Dutch (Caucasian) individuals (200 chromosomes) was also screened.

#### Plasmids

The human Na_v_1.7-AL insert (carrying the adult exon 5, E5A, and Long loop1), converted to become TTX-R (hNa_v_1.7_R_/AL; designated WT hereinafter) by Y362S substitution [39], has been previously described [39]. The I228M mutation was introduced into WT using QuickChange XL II site-directed mutagenesis according to manufacturer recommendations (Stratagene).

The full-length inserts of the different clones were sequenced at the Howard Hughes Medical Institute/Keck Biotechnology Center at Yale University. Sequence analysis used BLAST (National Library of Medicine) and Lasergene (DNAStar, Madison, WI), and confirmed the inserts to be devoid of un-intended mutations.

#### Transient transfection of HEK293 cells

Transient transfections of the hNa_V_1.7 together with hβ1 and hβ2 constructs into HEK293 cells were performed using Optifect (Invitrogen) following the recommended protocol by manufacturer. Recordings were performed 20-30 hours after transfection.

#### Primary sensory neuron isolation and transfection

Dorsal root ganglia (DRG) and trigeminal ganglia from adult Sprague Dawley rat pups (P0-P5) were isolated and then cultured using the same protocol for both. Dissected ganglia were placed in ice-cold oxygenated complete saline solution (CSS), which contained (in mM) 137 NaCl, 5.3 KCl, 1 MgCl_2_, 25 sorbitol, 3 CaCl_2_, 10 *N*-2-hydroxyethylpiperazine-*N'*-2-ethanesulfonic acid (HEPES); pH 7.2. They were then transferred to an oxygenated, 37°C CSS solution containing 1.5 mg/ml Collagenase A (Roche Applied Science, Indianapolis, IN) and 0.6 mM EDTA and incubated with gentle agitation at 37°C for 20 min. This solution was then exchanged with an oxygenated, 37°C CSS solution containing 1.5 mg/ml Collagenase D (Roche Applied Science, Indianapolis, IN), 0.6 mM EDTA and 30 U/ml papain (Worthington Biochemical, Lakewood, NJ) and incubated with gentle agitation at 37°C for 20 min. The solution was then aspirated and the ganglia triturated in DRG media (DMEM/Fl2 (1:1) with 100 U/ml penicillin, 0.1 mg/ml streptomycin (Invitrogen, Carlsbad, CA) and 10% fetal calf serum (Hyclone, Logan, UT), which contained 1.5 mg/ml bovine serum albumin (Sigma-Aldrich, St. Louis, MO) and 1.5 mg/ml trypsin inhibitor (Roche Applied Science, Indianapolis, IN).

Either WT or I228M variant channels were transiently transfected into the DRG or trigeminal ganglion neurons, along with enhanced-GFP, by electroporation with a Nucleofector II (Amaxa, Gaithersburg, MD) using Rat Neuron Nucleofector Solution and program G-013, as described previously [39]. The ratio of sodium channel to GFP constructs was 10:1. The transfected neurons were allowed to recover for 5 minutes at 37°C in 0.5 ml of Ca^2+^-free DMEM containing 10% fetal calf serum. The cell suspension was then diluted with DRG media containing 1.5 mg/ml bovine serum albumin and 1.5 mg/ml trypsin inhibitor, 80 μl was plated on 12 mm circular poly-D-lysine/laminin precoated coverslips (BD Biosciences, Bedford, MA) and the cells incubated at 37°C in 5% CO_2 _for 30 min. DRG media (1 ml/well), supplemented with 50 ng/ml each of mNGF (Alomone Labs, Jerusalem, Israel) and GDNF (Peprotec, Rocky Hill, NJ), was then added and the cells maintained at 37°C in a 5% CO2 incubator.

#### Electrophysiology

Whole-cell voltage-clamp recordings in HEK293 cells were carried out at 20 ± 1°C using a peltier temperature controller of the recording chamber. The extracellular solution contained (in mM): 140 NaCl, 3 KCl, 1 MgCl_2_, 1 CaCl_2_, and 10 HEPES, pH 7.3 with NaOH (adjusted to 320 mOsm with dextrose). The pipette solution contained (in mM): 140 CsF, 10 NaCl, 2 MgCl_2_, 1 EGTA, 10 HEPES, pH 7.3 with CsOH (adjusted to 310 mOsm with dextrose). Patch-pipettes had a resistance of 1-3 MΩ when filled with pipette solution. The calculated junction potential (JPcalc included in pCLAMP software) of 9 mV was not compensated. Upon achieving the whole-cell recording configuration, the pipette and cell capacitance were manually minimized using the Axopatch 200B (Molecular Devices, Union City, CA) compensation circuitry. To reduce voltage errors, 80-90% series resistance and prediction compensation was applied. Cells were excluded from analysis if the predicted voltage error exceeded 3 mV. The recorded currents were digitized at a rate of 50 kHz after passing through a low-pass Bessel filter setting of 10 kHz. The Axopatch 200B data were digitized using pCLAMP software (version 10) and a Digidata 1440A interface (Molecular Devices). Linear leak and residual capacitance artifacts were subtracted out using the P/N method. The Na^+ ^current recordings were initiated after a 5 minute equilibration period once whole-cell configuration was achieved.

Data analysis was performed using Clampfit (Molecular Devices) and Origin (Microcal Software, Northhampton, MA). To generate activation curves, cells were held at -120 mV and stepped to potentials of -80 to 40 mV for 100 msec. Peak inward currents obtained from activation protocols were converted to conductance values using the equation, G = I/(V_m _- E_Na_), for which G is the conductance, I is the peak inward current, V_m _is the membrane potential step used to elicit the response and E_Na _is the reversal potential for sodium (determined for each cell using the x-axis intercept of a linear fit of the peak inward current responses). Conductance data were normalized by the maximum conductance value and fit with a Boltzmann equation of the form G = G_min _+ (G_max_-G_min_)/(1 + exp[(V_1/2 _-V_m_)/k)], where V_1/2 _is the midpoint of activation and k is a slope factor. The kinetics of inactivation were assessed by fitting the falling phase of the currents with a single exponential function. To generate steady-state fast-inactivation curves, cells were stepped to inactivating potentials of -140 to 10 mV for 500 msec followed by a 20 msec step to -10 mV. The protocol for slow-inactivation consisted of a 30 second step to potentials varying from -120 to 10 mV, followed by a 100 msec step to -120 mV to remove fast-inactivation and a 20 msec step to -10 mV to elicit a test response. Peak inward currents obtained from steady-state fast-inactivation and slow-inactivation protocols were normalized by the maximum current amplitude and fit with a Boltzmann equation of the form I = I_min _+ (I_max_-I_min_)/(1 + exp[(V_m _- V_1/2_)/k)], where V_m _represents the inactivating pre-pulse membrane potential and V_1/2 _represents the midpoint of inactivation. For deactivation the cells were held at -120 mV and tail currents were generated by a brief 0.5 ms depolarization to -20 mV followed by a series of repolarizations ranging from -120 to -40 mV. The closing rate of the channels was obtained by fitting the tail currents with a single exponential function.

Whole-cell current-clamp recordings from isolated DRG or trigeminal ganglion neurons were performed using the Axopatch 200B amplifier, digitized using the Digidata 1440A interface and controlled using pCLAMP software. The bath solution for current-clamp recordings contained (in mM): 140 NaCl, 3 KCl, 2 MgCl_2_, 2 CaCl_2_, and 10 HEPES, pH 7.3 with NaOH (adjusted to 315mOsm with dextrose). The pipette solution contained (in mM): 140 KCl, 0.5 EGTA, 5 HEPES, and 3 Mg-ATP, pH 7.3 with KOH (adjusted to 300 mOsm with dextrose). The junction potential between these two solutions given by JPcalc was 5 mV but no correction was applied for current-clamp experiments. Recordings were performed on transfected presumptive nociceptive neurons based on the morphology of small diameter (20-28 μm) round cell bodies that also exhibited GFP fluorescence. All recordings were performed between 40 hr and 50 hr post-transfection 20 ± 1°C. Coverslips were transferred to a perfusable chamber (Warner Instruments, Hamden, CT) and all recordings were initiated within an hour. Whole-cell configuration was obtained in voltage-clamp mode before proceeding to the current-clamp recording mode. Cells with stable (< 10% variation) resting membrane potentials (RMPs) more negative than -35 mV and overshooting action potentials (> 85 mV RMP to peak) were used for further data collection. Input resistance was determined by the slope of a line fit to hyperpolarizing responses to current steps of 10-35 pA. Threshold was determined by the first action potential elicited by a series of depolarizing current injections that increased in 5 pA increments. The number of action potentials elicited in response to depolarizing current injections of 500 msec duration was also measured. After-hypolarization currents, and amplitude and width of action potentials were not formally analyzed in this study. Data are expressed as means ± standard error (SEM). Statistical significance was determined by Student's t-test, Mann-Whitney test (firing frequency) or *z*-test (frequency of spontaneous firing).

## Competing interests

The authors declare that they have no competing interests.

## Authors' contributions

ME acquired electrophysiological data, completed data analysis, and participated in writing the manuscript. CH acquired electrophysiological data, completed data analysis, and participated in writing the manuscript. JSC acquired electrophysiological data and completed data analysis. JGJH provided clinical assessment of patients. GL participated in study design and manuscript editing. JPHD provided genomic assessment of patients. MMG provided genetic analysis of patients. SDDH participated in study design, data analysis and manuscript editing. CGF provided overall project management, participated in study design, and writing of the manuscript. ISJM provided overall project management, participated in study design, and writing of the manuscript. SGW provided overall project management, participated in study design, data analysis, and writing of the manuscript. All authors have read and approved the final manuscript.
